# Folic Acid-Modified Fluorescent-Magnetic Nanoparticles for Efficient Isolation and Identification of Circulating Tumor Cells in Ovarian Cancer

**DOI:** 10.3390/bios12030184

**Published:** 2022-03-21

**Authors:** Yue Pan, Zhili Wang, Jialing Ma, Tongping Zhou, Zeen Wu, Pi Ding, Na Sun, Lifen Liu, Renjun Pei, Weipei Zhu

**Affiliations:** 1 Department of Gynecology and Obstetrics, The Second Affiliated Hospital of Soochow University, Suzhou 215004, China; ypan2020@sinano.ac.cn (Y.P.); jlma2021@sinano.ac.cn (J.M.); tpzhou2021@sinano.ac.cn (T.Z.); ezwu2019@sinano.ac.cn (Z.W.); liulifen1981@126.com (L.L.); 2 CAS Key Laboratory for Nano-Bio Interface, Suzhou Institute of Nano-Tech and Nano-Bionics, Chinese Academy of Sciences, Suzhou 215123, China; zlwang2013@sinano.ac.cn (Z.W.); pding2017@sinano.ac.cn (P.D.); nsun2013@sinano.ac.cn (N.S.)

**Keywords:** circulating tumor cells, ovarian cancer, folic acid, fluorescent-magnetic nanoparticles, isolation, identification

## Abstract

Ovarian cancer (OC) is a lethal disease occurring in women worldwide. Due to the lack of obvious clinical symptoms and sensitivity biomarkers, OC patients are often diagnosed in advanced stages and suffer a poor prognosis. Circulating tumor cells (CTCs), released from tumor sites into the peripheral blood, have been recognized as promising biomarkers in cancer prognosis, treatment monitoring, and metastasis diagnosis. However, the number of CTCs in peripheral blood is low, and it is a technical challenge to isolate, enrich, and identify CTCs from the blood samples of patients. This work develops a simple, effective, and inexpensive strategy to capture and identify CTCs from OC blood samples using the folic acid (FA) and antifouling-hydrogel-modified fluorescent-magnetic nanoparticles. The hydrogel showed a good antifouling property against peripheral blood mononuclear cells (PBMCs). The FA was coupled to the hydrogel surface as the targeting molecule for the CTC isolation, held a good capture efficiency for SK-OV-3 cells (95.58%), and successfully isolated 2–12 CTCs from 10 OC patients’ blood samples. The FA-modified fluorescent-magnetic nanoparticles were successfully used for the capture and direct identification of CTCs from the blood samples of OC patients.

## 1. Introduction

Ovarian cancer (OC) is the major lethal disease occurring in women worldwide [1]. Primary treatment of OC consists of cytoreductive surgery followed by chemotherapy [2]. The surgery cannot completely remove the tumor that has metastasized. Chemotherapy resistance and recurrence are common, resulting in impaired survival [3]. Thus, a high specificity and sensitivity method for cancer monitoring after treatment is desperately needed. Circulating tumor cells (CTCs) are a kind of cancer cell that circulate in the bloodstream after being released from solid tumors. These cells hold high metastatic ability and can cause tumor metastasis in remote locations [4,5,6,7]. CTCs have promising potential in monitoring the tumor prognosis, treatment effect, and metastasis condition [8,9,10]. However, due to the rare existence of CTCs in peripheral blood, the isolation and identification of variable CTCs is still challenging [11,12,13]. Epithelial to mesenchymal transition (EMT) plays a significant role during the CTCs’ metastatic process. It results in a phenotypic change in CTCs, such as transforming of epithelial properties to mesenchymal properties, and poses hurdles in CTC isolation [14,15]. Currently, many approaches have been devoted to isolating or detecting CTCs from blood samples, such as immunomagnetic separation, microfluidics, label-free CTC capture, electrochemistry, and fluorescence sensor [16,17,18,19,20,21,22,23,24,25]. Cancer-specific biomarkers such as Epithelial Cell Adhesion Molecule (EpCAM), N-cadherin, and Human Epidermal Growth Factor Receptor 2 (HER2) are overexpressed on the CTCs’ surface. Among them, antibody-dependent CTC enrichment using anti-EpCAM antibody has been mainly employed for CTC separation [16,17,18,19]. Our group has fabricated several antibody-modified interfaces and applied them to detect CTCs [26,27]. Still, the high cost of antibodies limits their practical use and underscores low-cost recognition molecules [28,29,30]. Folic acid (FA), a small molecule affinity agent specifically targeting the folate receptor (FR), attracts our attention due to its low cost, high affinity, and less immunogenicity [29,30]. The FR has been reported to be overexpressed on the cells’ surface of 90% of ovarian carcinomas, while few expressed in most normal tissues [31,32]. It was considered a good choice for CTC isolation of OC patients.

**Figure 1:**
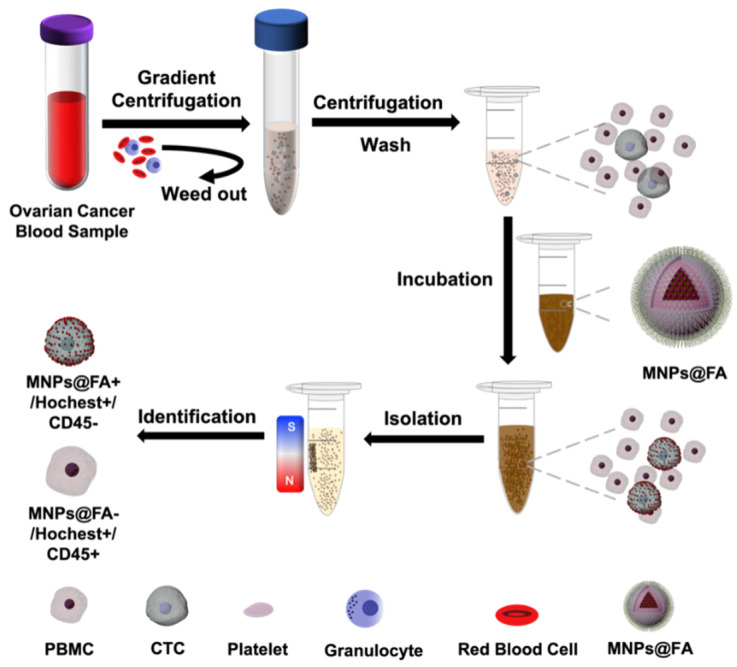
Schematic illustration of the process of CTC separation and identification by folic acid (FA)-modified fluorescent-magnetic nanoparticles (MNPs@FA).

The fluorescence probe is a powerful technology for cell biology research because of its simplicity, noninvasiveness, and real time. Herein, CdSe/ZnS quantum dots (QDs) were assembled onto the surface of Fe_3_O_4_ nanoparticles through electrostatic attraction, zwitterionic poly(sulfobetaine methacrylate) (pSBMA) was applied to overcome the nonspecific adhesion of blood cells, and finally, FA was modified on the nanoparticles’ surface for the specific capture of CTCs. The clinical blood samples of OC patients were used to evaluate the potential ability to practice the platform for CTC capture and identification. The schematic process of CTC separation and identification using FA-modified fluorescent-magnetic nanoparticles was outlined in Figure 1. Overall, the results demonstrated that FA-modified fluorescent-magnetic nanoparticles offer a cost-effective, reliable, and noninvasive method for the quick detection and identification of CTCs in patients with ovarian cancer.

## 2. Materials and Methods

### 2.1. Reagents and Cells Culture

Iron chloride hexahydrate (FeCl_3_·6H_2_O), trisodium citrate dihydrate, ammonium acetate, ethylene glycol (EG), ethanol, ammonia aqueous, tetraethyl orthosilicate (TEOS), and acetonitrile were purchased from Sinopharm Chemical Reagent Co., Ltd. (Shanghai, China). The CdSe/ZnS quantum dots (QDs) were obtained from Xingzi New Material Technology Development Co., Ltd. (Shanghai, China). Polyethylenimine (PEI, branched, 25,000 Mw), 3-(trimethoxylsilyl) propyl methacrylate (MPS), N-(3-sulfopropyl)-N-(methacryloxyethyl)-N,N-dimethylammonium betaine (SBMA), methacrylic acid (MAA), N,N′-methylenebis(2-propenamide) (MBA), 2,2-azobisisobutyronitrile (AIBN), 1-ethyl-3-(3′-dimethylaminopropyl) carbodiimide (EDC), N-hydroxysuccinimide (NHS), Histopaque-1077 solution, Hoechst 33,342, 3-3′-dioctadecyloxa-carbocyanine perchlor (DiO), and 1,1′-dioctadecyl-3,3,3′,3′-tetramethylindocarbocyanine perchlo-rate (DiI) were purchased from Sigma-Aldrich (St. Louis, MO, USA). Alexa Fluor 488-modified anti-CD45 (CD45) and Alexa Fluor 555-modified anti-Pan-Keratin (PanCK) antibodies were obtained from Cell Signaling Technology, Inc. (Beverly, MA, USA). Amine-PEG_5000_-folic acid (NH_2_-PEG_5000_-FA) was prepared by our previous work [30]. RIPA lysis buffer was purchased from Beyotime Biotechnology (Shanghai, China). Protease inhibitor cocktail was obtained from Roche (Mannheim, Germany). BCA protein assay kit was purchased from UU Biotechnology Co., Ltd. (Suzhou, China). FORL1 mouse monoclonal antibody (anti-α-FR), anti-β-actin antibody, and HRP-conjugated secondary antibody (anti-mouse) were purchased from Proteintech (Chicago, IL, USA).

The ovarian adenocarcinoma cell lines (SK-OV-3 and OVCAR-3) and human embryonic kidney cell lines (293T) were provided by the Cell Resource Centre of Life Sciences (Shanghai, China), and the ovarian cancer A2780 cell line was obtained from the Soochow University. The cells culture complied with the instructions of the Cell Resource Centre and the friendship provider.

### 2.2. Preparation of FA-Modified Fluorescent-Magnetic Nanoparticles

#### 2.2.1. Synthesis of Fe_3_O_4_ Nanoparticles (MNPs)

Fe_3_O_4_ magnetic nanoparticles (MNPs) were synthesized according to the reported methods [33]. Briefly, FeCl_3_·6H_2_O (1.350 g), ammonium acetate (3.854 g), and trisodium citrate dihydrate (0.4 g) were added into EG (70 mL) and stirred until they turned to a brown homogeneous mixture. The solution was heated to 170 °C for 1 h before being transferred to a 100 mL Teflon-lined stainless-steel autoclave. After heating at 200 °C for 16 h, the autoclave was cooled to room temperature and the black Fe_3_O_4_ magnetic nanoparticles were separated from the solution by using the magnet. After washing with ethanol and deionized water several times, the product was dispersed in ultrapure water and quantified for subsequent use.

#### 2.2.2. Preparation of Fluorescent-Magnetic Nanoparticles (MNPs@QD)

MNPs (200 mg) were dispersed in 40 mL of deionized water containing 200 mg of PEI. The mixture was stirred for 2 h and after washing with water, PEI-modified MNPs (MNPs@PEI) were re-dissolved in 20 mL of deionized water, and then 0.5 mg of quantum dots were added into the solution with stirring overnight. Finally, the fluorescent-magnetic nanoparticles (MNPs@QD) were obtained and protected from light.

#### 2.2.3. Fabrication of Functionalized Hydrogel MNPs (MNPs@hydrogel)

MNPs@QD (50 mg) was dispersed in ethanol (40 mL) and ultrapure water (10 mL) under the ultrasonic condition for 10 min. Next, ammonia solution (0.5 mL) was added into the mixture, and then TEOS (0.25 mL) was dropped into the solution with mechanical stirring at room temperature for 6 h under dark conditions. The obtained product (MNPs@Si) was separated by a magnet and washed with ethanol several times.

The synthesized MNPs@Si (25 mg) was dissolved in the solution consisting of ethanol (50 mL) and MPS (0.5 mL). After reacting for 48 h at room temperature, the product was dried at 40 °C and then re-dissolved in the acetonitrile (80 mL) containing SBMA (1.35 mg), MBA (100 mg), AIBN (10 mg), and MAA (100 mL). Then, the mixture was refluxed at 110 °C for 1 h. After washing, the hydrogel-coated MNPs (MNPs@hydrogel) were resuspended in ultrapure water and quantified for further use.

#### 2.2.4. Synthesis of MNPs@FA

Briefly, the MNPs@hydrogel particles were activated with 0.2 M of EDC and 0.05 M of NHS in 1 mL of MES at room temperature with gentle shaking for 2 h, immediately followed by the reaction with NH_2_-PEG_5000_-FA for at least 24 h in dark conditions at room temperature. After being washed by phosphate buffered solution (PBS) with the help of a magnetic scaffold, unanchored NH_2_-PEG_5000_-FA molecules were removed, and MNPs@FA was obtained for further application.

### 2.3. Characterization of Nanoparticles and Captured Cells

The morphologies, average size, mono-dispersibility, zeta potential, and fluorescent images of nanoparticles were separately characterized by a transmission electron microscope (TEM, 120 kV, Hitachi-HT7700), a thermal field-emission environmental scanning electron microscope (SEM, 20.0 kV, FEI Quanta 400F), a Zetasizer Nano ZS (Malvern Instruments, Malvern, UK) and a confocal microscope (Olympus FV500-IX81, Tokyo, Japan). Optical density (OD) was measured by microplate reader (Perkin Elmer VICTORTM X4, Waltham, MA, USA). Western blot chemiluminescent signals were detected by the ECL Western blot detection system (Cwbiotech, Beijing, China). The fluorescence signal of the samples was measured by C6 cytometer (BD Biosciences, Ann Arbor, MI, USA). The SEM was also used to image the cells after 4% of paraformaldehyde fixation and gradient ethanol dehydration (30%, 50%, 70%, 85%, 95%, and 100%). The fluorescent images of the captured SK-OV-3 cells were observed by the confocal microscope after fixing with paraformaldehyde (4%) and stained with Hoechst 33,342 and Alexa Fluor 488-modified anti-CD45 or Alexa Fluor 555-modified anti-Pan-Keratin.

### 2.4. Exploration of Capture Performance

#### 2.4.1. Investigation of Optimum Capture Conditions

The different doses of MNPs@FA (0.04 mg, 0.08 mg, 0.12 mg, 0.16 mg, 0.20 mg, 0.24 mg) were used to incubate with 1.0 × 10^5^ of SK-OV-3 cells in 1 mL of the cell culture medium in 1.5 mL of EP tubes for 30 min at 37 °C to determine the dose-dependent effect of MNPs@FA on their capture efficiency. The cells captured by MNPs@FA were collected and washed by the magnetic scaffold and PBS. Finally, the cell samples were counted by a hemocytometer and a microscope. In addition, the capture efficiency was affected by the modification concentrations of NH_2_-PEG_5000_-FA (0.01, 0.05, 0.1, 0.15, 0.2, 0.25 mg) and the capturing time (5, 10, 15, 20, 25, 30 min). Furthermore, the capture yields of the differently modified magnetic nanoparticles were also researched using the discussed method.

#### 2.4.2. Flow Cytometric Analysis and Western Blotting

For flow cytometric analysis, the SK-OV-3, OVCAR-3, A2780, and HEK293T cells were treated into suspension, then these cells and extracted PBMCs were incubated with FORL1 mouse monoclonal antibody (diluted 1:1000) for 30 min on ice. After washing, the cells were re-suspended in 100 µL of PBS, incubated with FITC-labeled goat anti-mouse antibody (diluted 1:1000) for 30 min on ice. Cells were then washed and analyzed by flow cytometer. The whole cells were lysed with RIPA lysis buffer supplemented with a protease inhibitor cocktail, and the lysates were clarified by centrifugation. The lysates were determined by the BCA protein assay kit, and separated by sodium dodecyl sulfate polyacrylamide gel electrophoresis (SDS-PAGE) at 100 V for 2 h, and then transferred to a polyvinylidene difluoride (PVDF) membrane at 100 V for 1.5 h. After blocking in 5% powdered milk, the membranes were probed with anti-FR antibody and anti-β-actin antibody, followed by HRP-conjugated secondary antibody. The chemiluminescent signals were detected by the ECL Western blot detection system.

#### 2.4.3. Verification of Capture Specificity

The nanoparticles were incubated with 1.0 × 10^5^ of SK-OV-3 cells (high-expressing FR), OVCAR-3 (high-expressing FR), A2780 cells (high-expressing FR), 293T cells (low-expressing FR), and peripheral blood mononuclear cell (PBMCs, separated from healthy blood samples, low-expressing FR) under the optimum capture condition, to determine the captured specificity of DiI pre-stained SK-OV-3 cells (1.0 × 10^5^) and DiO pre-stained PBMCs (1.0 × 10^5^) were mixed in the EP tube. Then, the mixed cells were incubated with the MNPs@FA under the optimum conditions to further verify the capture specificity. The fluorescence microscope enumerated the captured cells.

#### 2.4.4. Cell Viability Analyses of Captured Tumor Cells

The SK-OV-3 cells viabilities captured by MNPs@FA (no modification of QD) and original SK-OV-3 cells (cells before capturing by MNPs@FA) were monitored through staining with 2 μm calcein-AM (green, live cells) and 4.5 μm PI (red, dead cells). The results were counted by fluorescence microscope. Furthermore, the cytotoxicity of MNPs@FA in vitro was analyzed by the CCK-8 assay. Briefly, the SK-OV-3 cells were each seeded into 96-well plates at the density of 5 × 10^3^ cells/well and cultured for 24 h. Washed the cells thrice with PBS, 100 μL fresh medium containing different concentrations of MNPs@FA (0, 0.05, 0.1, 0.2, 0.4 mg/mL) was added per well. After culturing for 24 h, 10 μL CCK-8 was added to each well, and the cells were incubated further for 2 h. Measured optical density (OD) of the cell suspensions at 450 nm by microplate reader (Perkin Elmer VICTORTM X4). The cell viability was calculated as OD sample/OD control ×100%, where sample refers to the treated cells and control refers to the untreated cells.

#### 2.4.5. Capture of Rare Cells

The artificial samples were prepared by spiking DiI pre-stained SK-OV-3 cells (5, 10, 50, 100, 200) into 1 mL of PBS or pre-treated whole blood from healthy people. The samples were separately incubated with MNP@FA (0.2 mg/mL) for 25 min. The mixtures were then placed on the magnetic separator for 2 min to isolate the captured cells by MNPs@FA, respectively. After washing by PBS, the fluorescence microscope enumerated capture cells to evaluate the capture yields. Meanwhile, the unstained SK-OV-3 cells (5, 10, 50, 100, 200) in the pre-treated whole blood were also captured by MNP@FA (0.2 mg/mL) for 25 min and then counted by the fluorescence microscope under the autofluorescence of magnetic nanoparticles to assess the capture yields.

### 2.5. CTC Isolation of Ovarian Cancer Patient Peripheral Blood Samples

The whole blood samples (*n*, 10) from OC patients of the Second Affiliated Hospital of Soochow University were collected and kept in ethylenediaminetetraacetic acid (EDTA) vacutainer tubes. This study was approved by the ethics review board of the Second Affiliated Hospital of Soochow University (Approval # JD-LS-2019-090-01). All blood samples were pretreated via gradient centrifugation to collect PBMCs containing CTCs. The isolation of CTCs was performed according to the same procedure as the artificial samples. After isolating and washing, the captured cells were diluted in 4% paraformaldehyde (PFA) and fixed on an adhesion slide, and then blocked by Triton X-100 (0.3%) and BSA (1%) for 1 h. The samples were stained with Alexa Fluor 488-modified anti-CD45 and/or Alexa Fluor 555-modified anti-Pan-Keratin overnight. Afterward, the samples were mixed with Hoechst 33,342 for 15 min and washed with water several times. Finally, the cells were observed and enumerated by the confocal microscope. Cells that displayed Hoechst 33,342+/MNPs@FA+/CD45− or Hoechst 33,342+/MNPs@FA+/PanCK+/CD45− with morphologically intact were identified as CTCs.

## 3. Results and Discussion

### 3.1. Preparation and Characterization of MNPs@FA

**Figure 2:**
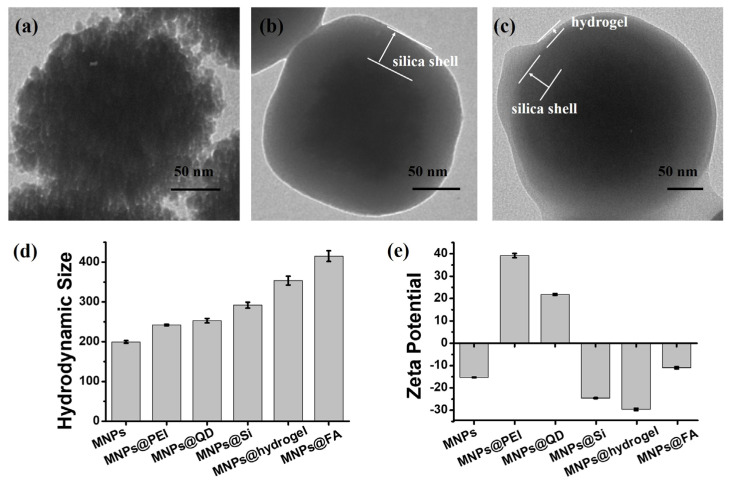
Transmission electron microscope (TEM) images of (**a**) MNPs@QD, (**b**) MNPs@Si, and (**c**) MNPs@hydrogel. Comparison of hydrodynamic size (**d**) and zeta potential (**e**) by the DLS and zeta potential measurement for the nanoparticles with different modifications. All data are expressed as the mean ± stand deviation, *n* = 3.

The synthesis of MNPs@FA was outlined in Figure S1. In brief, magnetic nanoparticles (MNPs) were synthesized by the solvothermal method. As displayed in Figure S2a, MNPs showed a good uniformity in size with a diameter of about 180 nm. Next, the PEI was modified on the surface of the magnetic nanosphere by electrostatic attraction (MNPs@PEI). As shown in Figure S2b, MNPs covered by a polymer layer tend to surface smooth of the nanoparticles. As shown in Figure 2a and Figure S2c,d, the QDs (10 nm) were further bedecked on the MNPs@PEI with the help of electrostatic interaction (MNPs@QD). The TEM images showed uniformity QD particles on the surface of MNPs@QD. Figure 2b and Figure S2e showed the MNPs@QD was coated in a silica shell (MNPs@Si). The silica thickness is about 45 nm. Based on our previous method [27], the antifouling hydrogel was formed on the surface MNPs@Si using SBMA, MAA, and MBA to ensure the purity and identification of CTC capture. Figure 2c and Figure S2f showed hydrogel shell (thickness, 12 nm) was successfully coated on the surface MNPs@Si. Meanwhile, the carboxyl group contained in the hydrogel facilitated further NH_2_-PEG_5000_-FA modification to achieve specific and efficient CTC capture. The hydrodynamic size and zeta potential of nanoparticles produced during the process were separately characterized, as shown in Figure 2d,e. The size of MNPs@PEI was significantly increased relative to the MNPs, and the potential of the nanoparticles was changed from a negative potential to a positive potential, which indicated that the PEI was successfully adsorbed on the surface of MNPs. After modifying the QDs, the size slightly increased because the surface of the QDs was modified with carboxyl groups, and the zeta potential of MNPs@QD was lower than that of MNPs@PEI. After further coating of silica shell and hydrogel, due to silicon hydroxyl, sulfonic group, and carboxyl groups, the potential of MNPs@Si and MNPs@hydrogel showed a negative value, and the size increased distinctly. After modifying NH_2_-PEG_5000_-FA, the carboxyl groups on the surface of MNPs@hydrogel were replaced by NH_2_-PEG_5000_-FA; the potential and size of MNPs@FA were visibly increased. The results demonstrated that products from MNPs to MNPs@FA were successfully synthesized.

**Figure 3:**
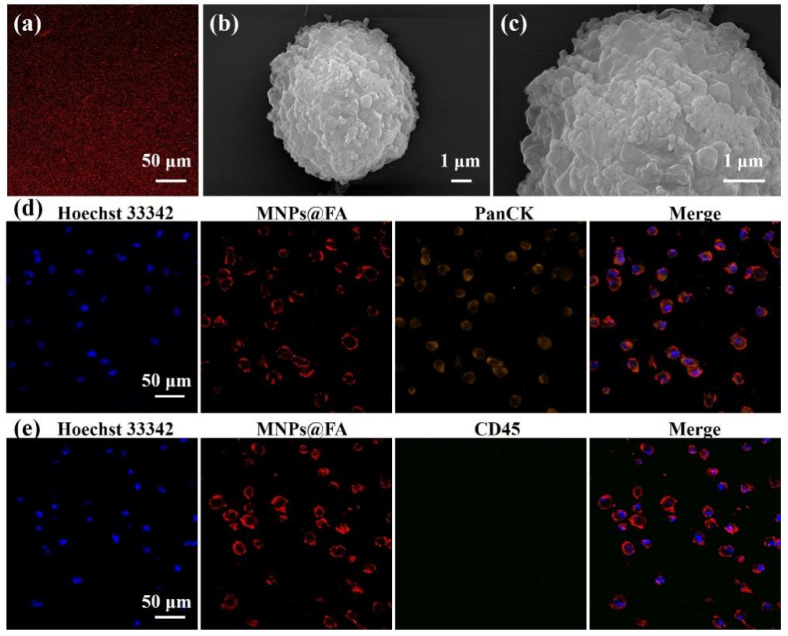
(**a**) A fluorescent image of MNPs@FA showing a stable fluorescent signal. (**b**,**c**) SEM images of an SK-OV-3 cell captured by MNPs@FA with a sufficient number of nanoparticles on the cell surface. (**d**) Fluorescent images of SK-OV-3 cells captured by MNPs@FA (red) with immunostaining of anti-PanCK-555 (orange) and Hoechst 33,342 (blue). (**e**) Fluorescent images of SK-OV-3 cells captured by MNPs@FA (red) with immunostaining of anti-CD45-488 (green) and Hoechst 33,342 (blue).

To further verify the capacity of MNPs@FA for CTC identification with fluorescence, the MNPs@FA nanoparticles were characterized by a confocal microscope. As shown in Figure 3a, the fluorescent image of MNPs@FA showed that the QDs were well modified into the silica shell, which ensured the fluorescent identification of MNPs@FA for target cells. As shown in Figure S3, SK-OV-3 cells were used as the model cells due to their high expression of folate receptors. SK-OV-3 cells captured by MNPs@FA observed by SEM were shown in Figure 3b,c; compared with original SK-OV-3 cells shown in Figure S4, an abundance of nanoparticles could be seen on the captured cell surface. Additionally, to confirm the feasibility of MNPs@FA for CTC identification, the fluorescence property of MNPs@FA on captured target cells was researched by observing SK-OV-3 cells under the confocal microscope. After staining by Hoechst 33,342 (blue) and anti-PanCK-555 (orange), as shown in Figure 3d, a clear red shell from MNPs@FA could be observed on the surface of the cells, which had a fine fluorescent consistency with immunostaining from anti-PanCK-555. In consideration of the presence of anti-CD45-488 used for PBMC identification in patient blood samples, the target cells were stained with MNPs@FA and anti-CD45-488 at the same time. As shown in Figure 3e, there was no cross-staining between the immunostaining of anti-CD45-488 and MNPs@FA on the captured target cells. The results further indicated that the MNPs@FA could provide the potential for CTC fluorescent identification. 

### 3.2. Cell Capture Performance of MNPs@FA

**Figure 4:**
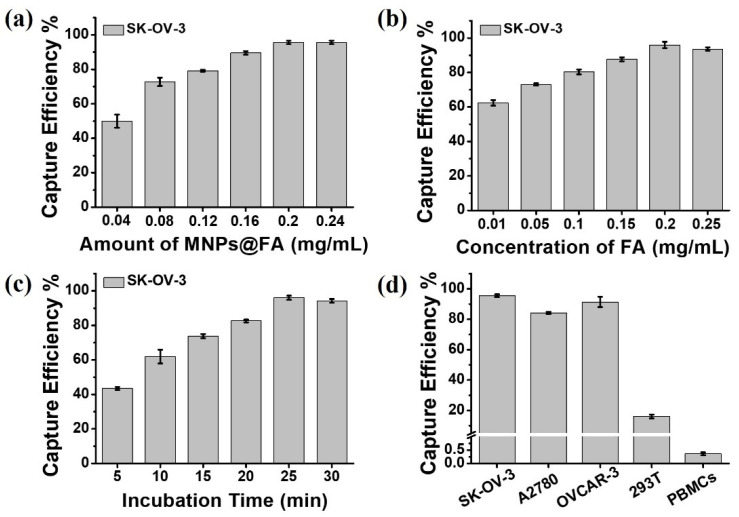
(**a**) Capture efficiency of MNPs@FA for SK-OV-3 cells at different concentrations (0.04, 0.08, 0.12, 0.16, 0.2, 0.24 mg/mL). (**b**) Capture efficiency of MNPs@FA for SK-OV-3 cells at the different modified concentrations of folic acid (0.01, 0.05, 0.1, 0.15, 0.2, 0.25 mg/mL). (**c**) Capture efficiency of MNPs@FA for SK-OV-3 cells at different incubation times (5, 10, 15, 20, 25, 30 min). (**d**) Capture efficiencies of MNPs@FA for different cells. All data are expressed as the mean ± stand deviation, *n* = 3.

The experimental conditions are crucial for CTC separation. A series of attempts were conducted to optimize the capture conditions of MNPs@FA with the help of SK-OV-3 cells. Firstly, the experiments were made to optimize the dosages of MNPs@FA, as shown in Figure 4a, 0.04 mg/mL of MNPs@FA showed a 49.92% of capture efficiency, when the dosage increased to 0.2 mg/mL, the capture efficiency increased to 95.58%, while 0.24 mg/mL of MNPs@FA brought a minor change to capture efficiency (95.67%). Therefore, 0.2 mg/mL of the MNPs@FA dosage was chosen for CTC capture. Furthermore, the different modified concentrations (0.01, 0.05, 0.1, 0.15, 0.2, 0.25 mg/mL) of folic acid were also investigated, as shown in Figure 4b, 0.2 mg/mL of FA brought a 96% capture efficiency, while with the addition of FA, the capture yield did not increase. Consequently, 0.2 mg/mL of FA-modified concentration was determined for CTC capture. Moreover, the incubation time was explored based on the above optimal conditions. As shown in Figure 4c, the capture efficiency reached 61.92% in the incubation time of 10 min. However, the capture efficiency rose sharply to 96.08% when the incubation time was 25 min. So, the 25 min incubation time was used for CTC capture.

We further explored the capture ability of different modified MNPs (MNPs@Si, MNPs@hydrogel, and MNPs@FA) for SK-OV-3 cells. Figure S5 summarized the capture yields of the different nanoparticles, and the results showed that the hydrogel formed by pSBMA possessed an excellent antifouling property. Meanwhile, the capture specificity of MNPs@FA was evaluated using FR-positive cancer cell lines SK-OV-3, A2780 and OVCAR-3, and FR-negative 293T cells and PBMCs from healthy donors (Figure S3) [34,35,36,37,38,39]. The capture specificity results shown in Figure 4d. The MNPs@FA had a fine capture efficiency for OVCAR-3 cells (95%) and A2780 cells (85%), and a low adhesion rate for 293T cells (16.17%) and PBMCs (0.39%). The results demonstrated that the FA modified MNPs could capture FR-positive CTCs both efficiently and specifically. Furthermore, the capture sensitivity of MNPs@FA was also investigated by spiking DiI prestained SK-OV-3 cells and DiO prestained PBMCs at a rate of 1:1 into 1 mL of PBS. As shown in Figure S6, the MNPs@FA exhibited a quite different isolation performance for the SK-OV-3 cells (94.25%) and PBMCs (0.41%), indicating an excellent capture specificity of MNPs@FA. In addition, the cell viability of captured SK-OV-3 cells was also assessed by live/dead staining. To ensure the count accuracy of live/dead fluorescence, MNPs without modifying QDs were used in the experiment. As shown in Figure S7, little difference between original cells and captured cells could be observed. The viability percentage of original cells was 97.97% and captured cells was 95.47%. Furthermore, we test the cytotoxicity of MNPs@ FA in the different concentration by the CCK-8 assay. The Figure S8 showed that the viability percentage of the SK-OV-3 cells incubated with 0.2 mg/mL dose of the MNPs@ FA nanoparticles for 24 h was more than 90%. These results indicated that MNPs@FA had little effect on the activity of captured cells.

### 3.3. Capture Sensitivity Test of MNPs@FA

**Figure 5:**
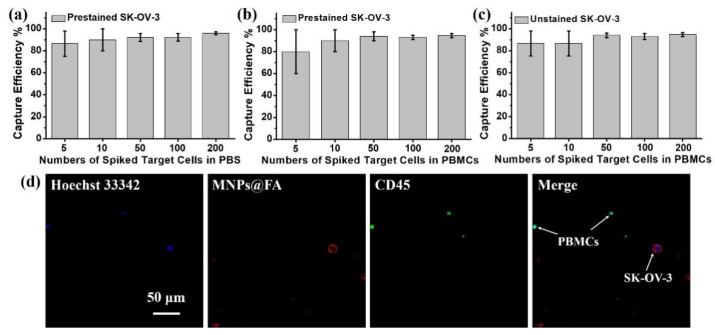
(**a**) The capture efficiency of MNPs@FA for rare DiI prestained SK-OV-3 cells in 1 mL of PBS. (**b**) The capture efficiency of MNPs@FA for rare DiI prestained SK-OV-3 cells in 1 mL of pre-treated PBMCs solution. (**c**) The capture efficiency of MNPs@FA for unstained SK-OV-3 cells in 1 mL of pre-treated PBMCs solution. All data are expressed as the mean ± stand deviation, *n* = 3. (**d**) Fluorescent images of MNPs@FA combined anti-CD45-488 and Hoechst 33,342+ for target cell and PBMCs.

To demonstrate the capture and identification capability of MNPs@FA for clinical samples, the rare number of DiI pre-stained SK-OV-3 cells (5, 10, 50, 100, 200) were spiked in 1 mL of PBS or pre-treated whole blood from healthy people (PBMCs). Meanwhile, we also spiked the same number of unstained SK-OV-3 cells in 1 mL of PBMCs solution and finished the counting for captured target cells based on the fluorescence performance of MNPs@FA. As shown in Figure 5a–c, the MNPs@FA showed a good capture efficiency for rare target cells, and the counting results by MNPs@FA fluorescence agreed well with the prestained counting group. Moreover, as shown in Figure 5d, the MNPs@FA combined anti-CD45-488 and Hoechst 33,342+ had a fine fluorescence distinction between target cells (Hoechst 33,342+/MNPs@FA+/CD45−) and PBMC (Hoechst 33,342+/CD45−). The results demonstrated that MNPs@FA could provide a dependable identification and enumeration method of CTCs in blood samples. 

### 3.4. CTC Detection from OC Patient Blood Samples

**Figure 6:**
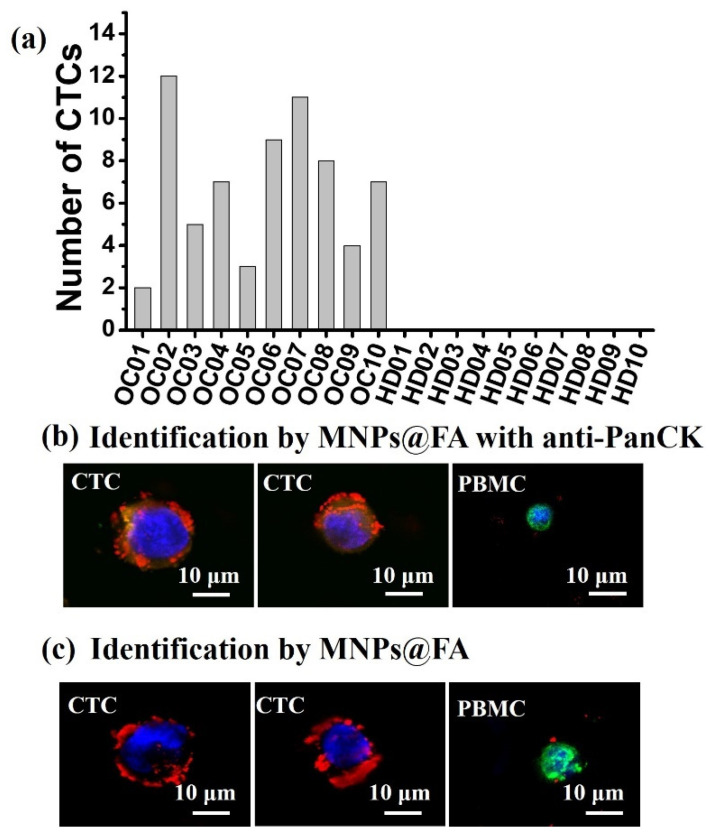
(**a**) Number of CTCs in the blood of 10 ovarian cancer patients (OC) and 10 healthy donors (HD) detected by the MNPs@FA. (**b**) Fluorescent images of CTCs and PBMCs identified by immunostaining of anti-PanCK-555 (orange) and anti-CD45-488 (green) as well as MNPs@FA (red) and Hoechst 33,342 (blue). (**c**) Fluorescent images of CTCs and PBMCs identified by Hoechst 33,342 (blue), anti-CD45-488 (green), and MNPs@FA (red).

The peripheral blood samples of ovarian cancer patients were provided by the Second Affiliated Hospital of Soochow University with patient-informed consent. The optimized capture parameters were applied to detect CTCs in the whole blood samples from 10 OC patients and 10 healthy donors (HD). The clinical characteristics of the blood samples were shown in Table S1. Figure 6a summarizes the CTC enumeration of OC patients and HD people. From 2–12 CTCs were detected from 3 mL of blood samples of 10 OC patients, whereas no CTCs were found in any of the healthy donors’ blood samples. Meanwhile, the immunostaining anti-PanCK were applied to identify the isolated CTCs from patient OC01 to validate the identification ability of MNPs@FA, and the same two CTCs were identified by the two identification methods (Figure 6b,c). The fluorescent images of CTCs and PBMCs identified by immunostaining of anti-PanCK-555, anti-CD45-488 and MNPs@FA for the sample OC01 were shown in Figure 6b. Besides, the fluorescent images of the CTCs identified by MNPs@FA and anti-CD45-488 without anti-PanCK-555 for the sample OC01 were displayed in Figure 6c. Cells that displaying Hoechst 33,342 (blue)+/MNPs@FA (red)−/PanCK(orange)−/CD45 (green)+ were counted as PBMCs, and showing Hoechst 33,342+/MNPs@FA+/CD45− or Hoechst 33,342+/PanCK+/MNPs@FA+/CD45− were counted as CTCs. The obtained results support that MNPs@FA hold powerful potential to capture and identify CTCs from patient’s blood samples.

## 4. Conclusions

This work developed a quick and effective method for the nondestructive and rapid capture and identification of CTCs using FA-modified fluorescent-magnetic nanoparticles. The incubation time might be different among different cell lines and different cancer types due to differences in biomarker expression levels. For exploring capture conditions in CTC capture, one cell line was used to be mainly chosen for obtaining the optimal incubation time in many pieces of research [28,40,41]. For example, Ding et al. built a simple and broad-spectrum method to efficiently isolate the heterogeneous CTCs from patient blood samples using tannic acid (TA)-functionalized magnetic nanoparticles (MNPs); the MCF-7 cell line was mainly chosen to explore the optimal capture conditions [28]; and Li et al. synthesized MN@Cys@PEG2k-FA magnetic nanospheres for early-stage cancer diagnosis targeted FR, which only chose the HeLa cell line for the experiment [41]. Therefore, in this work, SK-OV-3 cells with high FR expression were used as representative model cells to explore the relative optimal incubation time. A total of 0.2 mg MNPs@FA was incubated with 1 × 10^5^ SK-OV-3 cells at different incubation time. The optimal incubation time may depend on the targeting of MNPs@FA to the FR on SK-OV-3 cell surface to some extent. The capture efficiency rose to 96.08% when the incubation time was 25 min, and the 25 min incubation time was used for CTC capture in this work. Binding with folic acid, MNPs@FA held a good capture efficiency against FR-positive ovarian cancer cells (higher than 85%), while showing a low capture yield against PBMCs (0.39%). In addition, MNPs@FA exhibited a fine capture sensitivity in capturing four SK-OV-3 cells when five cells were spiked in PBMC solution separated from 1 mL of whole blood. In our study, 2–12 CTCs were detected from 3 mL blood samples of ovarian cancer patients. The large blood sample sizes may provide more chances in the capture number of CTCs from the blood samples of cancer patients. Moreover, the use of multiple trapping agents may increase the capture number for CTCs due to the presence of CTC heterogeneity. For example, the most typical representative and the only system approved by the U.S. Food and Drug Administration (FDA) in 2004, the CellSearch™ system, captures CTCs using the 7.5 mL peripheral blood sample [40]. However, most research detected CTCs in less than 7.5 mL blood samples. The nanocage-featured film successfully detected CTCs and CTC clusters in 2 mL or 4 mL blood taken from 21 cancer patients (stages I-IV) suffering from various types of cancers [24]. A PLGA nanofiber-based microfluidic device was fabricated with dual aptamer-targeting EpCAM and N-cadherin proteins, and 1 to 13 CTCs were successfully detected in 3 mL blood samples of ovarian cancer patients [34]. The FR is highly expressed on the surface of 90% of ovarian cancer cells and FA has a low cost and good specificity, therefore FA was chosen as a capture agent in this work for the CTC isolation of OC patients. However, the application of this method for the early detection of OC may have some limitations due to blood volume. To obtain more CTCs for downstream analysis, many efforts have been devoted to the CTC culture [42,43]. However, the optimization of CTC culture conditions will be needed before this strategy can be incorporated into clinical practice; therefore, more exploration and effort are needed on CTC in vitro culture studies. A few studies have been successful, including the CTC-derived pre-clinical model which consists of 2D cultures, the CTC-derived explant (CDX) model, and the 3D organoid generation; compared with other models, 3D culture has attracted the attention of scientists because of its advantages of stable morphology, gene expression, cell signaling, equal behavior and heterogeneity with cancer cells in the tumor mass, high throughput for drug screening, low cost, and easy operation “in a dish” [43]. In this study, MNPs@FA provided a reliable and noninvasive method for the quick detection and identification CTCs in ovarian cancer patients’ blood samples. Moreover, the high viability of captured cancer cells could further be used in in vitro cultures to help obtain more biological information of ovarian cancer patients in clinical applications.

## Data Availability

Not applicable.

## Supplementary Materials

The following supporting information can be downloaded at: https://www.mdpi.com/article/10.3390/bios12030184/s1, Figure S1: The interfacial modification of MNPs@FA for CTC isolation. Figure S2: Transmission electron microscope (TEM) images of (a) Fe_3_O_4_ nanoparticles (MNPs), (b) PEI-modified Fe_3_O_4_ nanoparticles (MNPs@PEI), (c) CdSe/ZnS quantum dots (QDs), (d) QDs-modified Fe_3_O_4_ nanoparticles (MNPs@QD), (e) silica-modified Fe_3_O_4_ nanoparticles (MNPs@Si) and (f) hydrogel-coated MNPs@Si (MNPs@hydrogel). Figure S3: The expression levels of α-FR protein in A2780, OVCAR-3, SK-OV-3, PBMCs, and HEK 293T cells analyzed by flow cytometry (a-e) and Western blots (f). Figure S4: Scanning electron microscopy (SEM) images of an SK-OV-3 cell without MNPs@FA nanoparticles on the cell surface (a,b). Figure S5: Comparison of capture efficiencies for SK-OV-3 cells by MNPs@Si, MNPs@hydrogel, and MNPs@FA (folic acid-modified MNPs@hydrogel). Figure S6: Capture performance of MNPs@FA for SK-OV-3 cells and PBMCs from the mixture sample at the ratio of 1:1. Figure S7: Cell viability of captured SK-OV-3 cells as well as original SK-OV-3 cells was evaluated by a live/dead staining. (a) Comparison of viability percentage of original and captured cells. Fluorescence imaging of cell viability of (b) original SK-OV-3 cells and (c) captured SK-OV-3 cells using live/dead staining (green: live; red: dead). Figure S8: The viability of SK-OV-3 cells incubated with different concentrations of MNPs@FA for 24 h. Table S1: Clinical information of ovarian cancer (OC) patients and healthy donors (HD) enrolled in this study.
