# Supplementary material for: Folic Acid-Modified Fluorescent-Magnetic Nanoparticles for Efficient Isolation and Identification of Circulating Tumor Cells in Ovarian Cancer
Source: Biosensors (Basel). 2022 Mar 21;12(3):184. doi: 10.3390/bios12030184 (PMC8946694; doi:10.3390/bios12030184)
Supplement: Supplementary file 1 [file biosensors-12-00184-s001.zip › biosensors-1556512-supplementary.pdf]

biosensors

Supplementary Materails

Folic Acid-Modified Fluorescent-Magnetic Nanoparticles for Efficient Isolation and Identification of Circulating Tumor Cells in Ovarian Cancer

Yue Pan [1,2], Zhili Wang [2], Jialing Ma [1,2], Tongping Zhou [1,2], Zeen Wu [1,2], Pi Ding [2], Na Sun [2], Lifen Liu [1], Renjun Pei [2,*] and Weipei Zhu [1,*]

[1] Department of Gynecology and Obstetrics, The Second Affiliated Hospital of Soochow University, Suzhou 215004, China; ypan2020@sinano.ac.cn (Y.P.); jlma2021@sinano.ac.cn (J.M.); tpzhou2021@sinano.ac.cn (T.Z.); ezwu2019@sinano.ac.cn (Z.W.); liulifen1981@126.com (L.L.)

[2] CAS Key Laboratory for Nano-Bio Interface, Suzhou Institute of Nano-Tech and Nano-Bionics, Chinese Academy of Sciences, Suzhou 215123, China; zlwang2013@sinano.ac.cn (Z.W.); pding2017@sinano.ac.cn (P.D.); nsun2013@sinano.ac.cn (N.S.)

* Correspondence: rjpei2011@sinano.ac.cn (R.P.); zwp333xx@126.com (W.Z.); Tel.: +86-0512-62872776 (RJ.P.); +86-0512-67784769 (W.Z.)

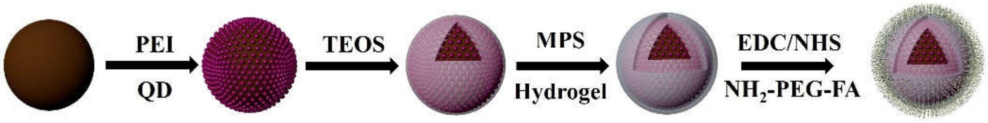

Figure S1. The interfacial modification of MNPs@FA for CTC isolation.

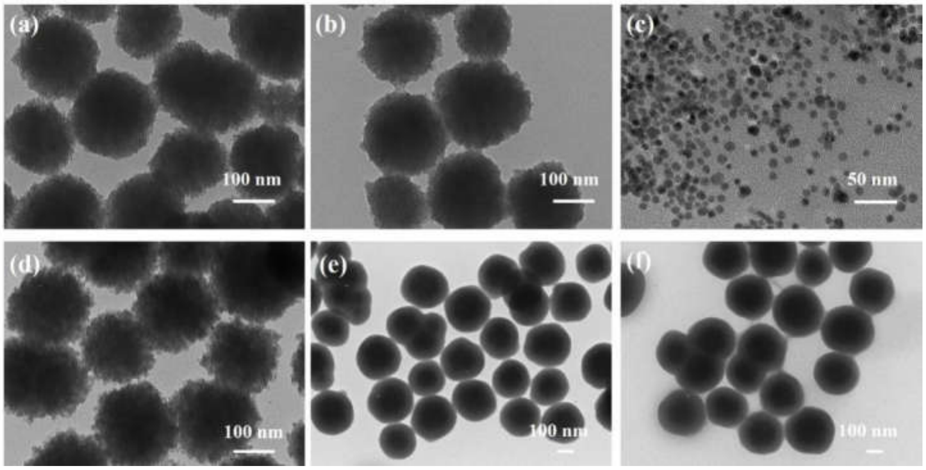

Figure S2. Transmission electron microscope (TEM) images of (**a**) Fe_3O_4 nanoparticles (MNPs), (**b**) PEI-modified Fe_3O_4 nanoparticles (MNPs@PEI), (**c**) CdSe/ZnS quantum dots (QDs), (**d**) QDs-modified Fe_3O_4 nanoparticles (MNPs@QD), (**e**) silica-modified Fe_3O_4 nanoparticles (MNPs@Si) and (**f**) hydrogel-coated MNPs@Si (MNPs@hydrogel).

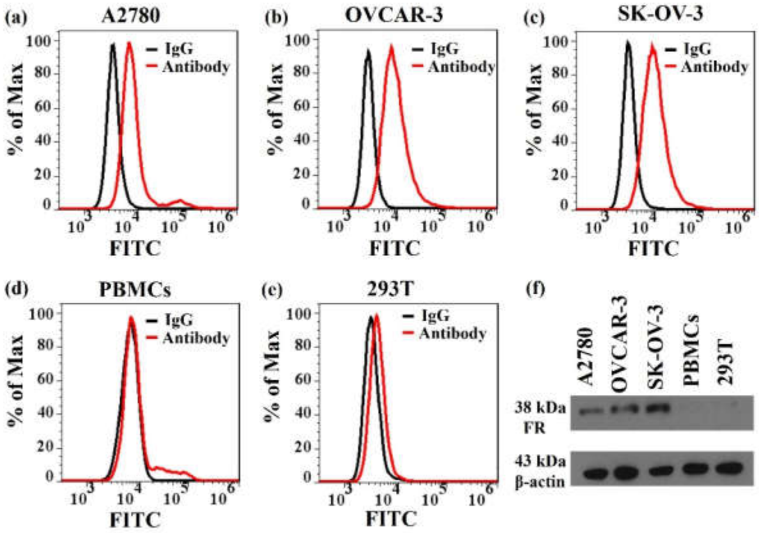

Figure S3. The expression levels of α-FR protein in A2780, OVCAR-3, SK-OV-3, PBMCs, and HEK 293T cells analyzed by flow cytometry (**a**–**e**) and western blots (**f**). The cell-surface overexpression of α-FR protein was confirmed by flow cytometry using anti-α-FR antibody, and the IgG was used as the control.

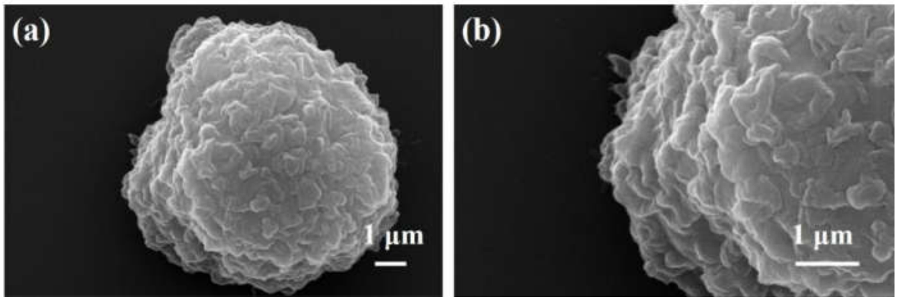

Figure S4. Scanning electron microscopy (SEM) images of an SK-OV-3 cell without MNPs@FA nanoparticles on the cell surface (**a**) and (**b**).

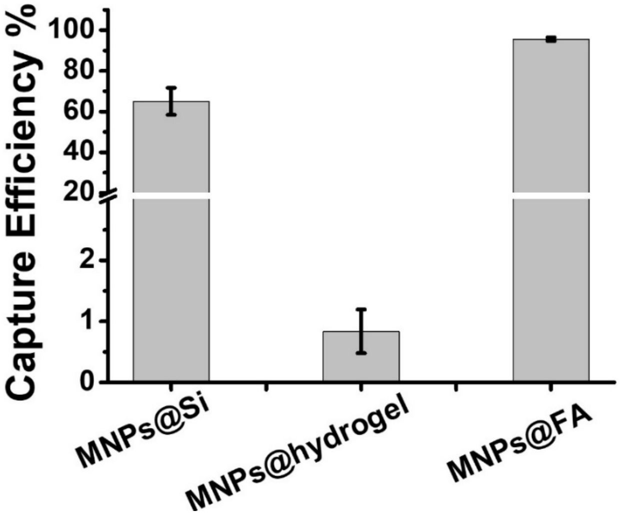

Figure S5. Comparison of capture efficiencies for SK-OV-3 cells by MNPs@Si, MNPs@hydrogel, and MNPs@FA (folic acid-modified MNPs@hydrogel).

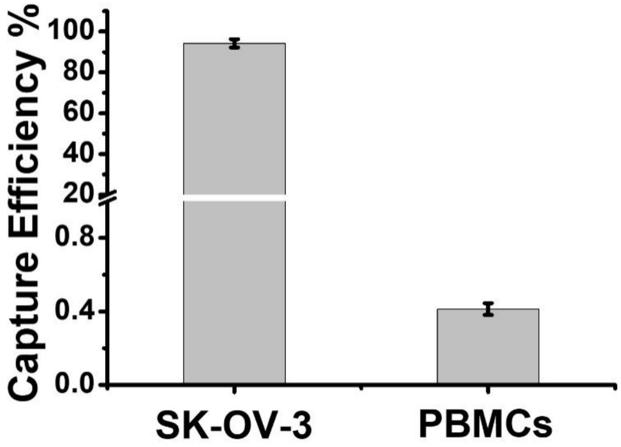

Figure S6. Capture performance of MNPs@FA for SK-OV-3 cells and PBMCs from the mixture sample at the ratio of 1:1.

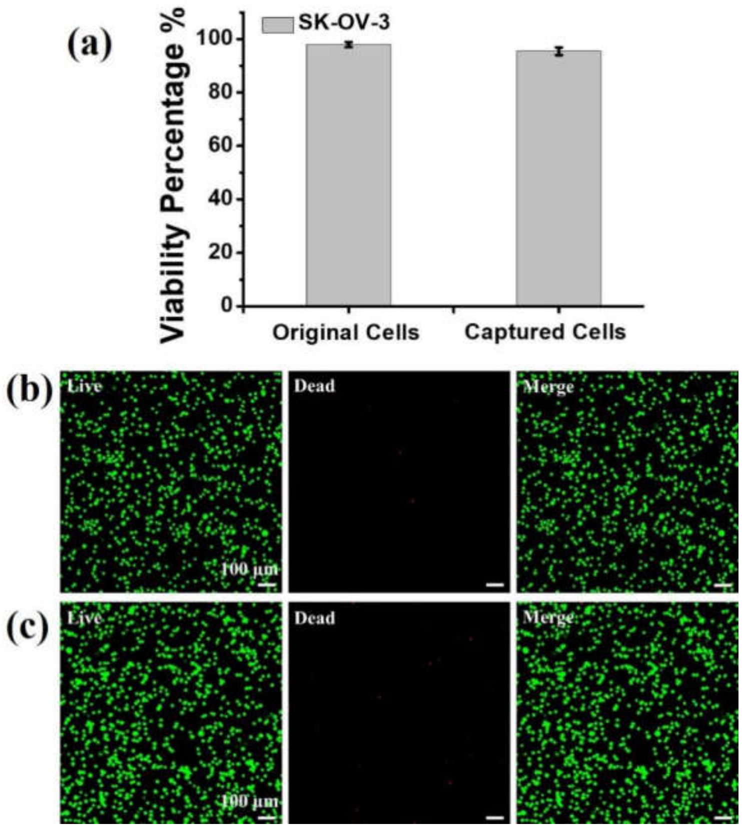

Figure S7. Cell viability of captured SK-OV-3 cells as well as original SK-OV-3 cells was evaluated by a live/dead staining. (**a**) Comparison of viability percentage of original and captured cells. Fluorescence imaging of cell viability of (**b**) original SK-OV-3 cells and (**c**) captured SK-OV-3 cells using live/dead staining (green: live; red: dead).

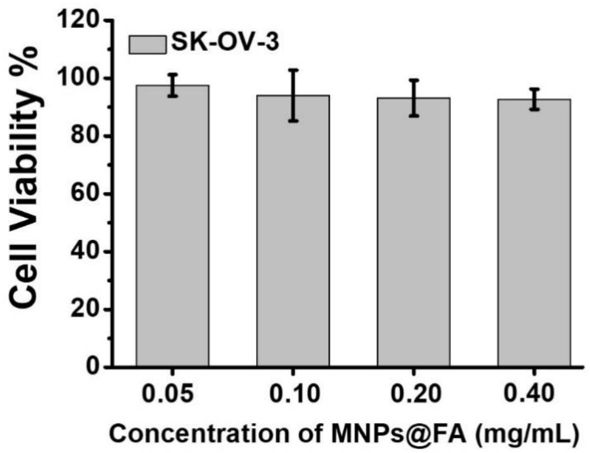

Figure S8. The viability of SK-OV-3 cells incubated with different concentrations of MNPs@FA for 24 h.

Table S1. Clinical information of ovarian cancer (OC) patients and healthy donors (HD) enrolled in this study.

Diagnosis	Patient no.	Age	Histology	FIGO	Therapy		Volume of blood (mL)	CTC counts
					Surgery	Chemo-therapy		
Ovarian cancer	OC01	58	EOC	IIIA	N	N	3	2
	OC02	74	EOC	IIIC	Y	Y	3	12
	OC03	58	EOC	IIIA	Y	N	3	5
	OC04	51	OSCST	IV	Y	Y	3	7
	OC05	53	EOC	III	Y	Y	3	3
	OC06	58	EOC	IIIC	Y	Y	3	9
	OC07	76	EOC	IIIC	Y	Y	3	11
	OC08	41	EOC	IIIC	Y	Y	3	8
	OC09	68	EOC	IIIC	Y	N	3	4
	OC10	32	EOC	IIIC	Y	Y	3	7
Healthy donors	HD01	26	N/A	N/A	N/A	N/A	3	0
	HD02	31	N/A	N/A	N/A	N/A	3	0
	HD03	47	N/A	N/A	N/A	N/A	3	0
	HD04	63	N/A	N/A	N/A	N/A	3	0
	HD05	52	N/A	N/A	N/A	N/A	3	0
	HD06	60	N/A	N/A	N/A	N/A	3	0
	HD07	49	N/A	N/A	N/A	N/A	3	0
	HD08	51	N/A	N/A	N/A	N/A	3	0
	HD09	48	N/A	N/A	N/A	N/A	3	0
	HD10	58	N/A	N/A	N/A	N/A	3	0
